# Refer-to-pharmacy: a qualitative study exploring the implementation of an electronic transfer of care initiative to improve medicines optimisation following hospital discharge

**DOI:** 10.1186/s12913-018-3262-z

**Published:** 2018-06-07

**Authors:** Jane Ferguson, Liz Seston, Darren M. Ashcroft

**Affiliations:** 1 Centre for Pharmacoepidemiology and Drug Safety, Division of Pharmacy & Optometry, School of Health Sciences, Manchester Academic Health Sciences Centre (MAHSC), University of Manchester, Oxford Road, Manchester, M13 9PT UK; 2 NIHR Greater Manchester Patient Safety Translational Research Centre, School of Health Sciences, University of Manchester, M13 9PL, Manchester, UK

**Keywords:** Normalisation process theory, Transfer of care, Medication errors, Electronic referral systems, Implementation

## Abstract

**Background:**

Transition between care settings is a time of high risk for preventable medication errors. Poor communication about medication changes on discharge from hospital can result in adverse drug events and medicines-related readmissions. Refer-to-Pharmacy is a novel electronic referral system that allows hospital pharmacy staff to refer patients from their bedside to their community pharmacist for post-hospital discharge medication support. The aim of this study was to examine factors that promoted or inhibited the implementation of Refer-to-Pharmacy in hospital and community settings.

**Methods:**

Twenty six interviews with hospital pharmacists (*n* = 11), hospital technicians (*n* = 10), and community pharmacists (*n* = 5) using Normalisation Process Theory (NPT) as the underpinning conceptual framework for data collection and analysis.

**Results:**

Using NPT to understand the implementation of the technology revealed that the participants unanimously agreed that the scheme was potentially beneficial for patients and was more efficient than previous systems (coherence). Leadership and initiation of the scheme was more achievable in the contained hospital environment, while initiation was slower to progress in the community pharmacy settings (cognitive participation). Hospital pharmacists and technicians worked flexibly together to deliver the scheme, and community pharmacists reported better communication with General Practitioners (GPs) about changes to patients’ medication (collective action). However, participants reported being unaware of how the scheme impacted patients, meaning they were unable to evaluate the effectiveness of scheme (reflexive monitoring).

**Conclusion:**

The Refer-to-Pharmacy scheme was perceived by participants as having important benefits for patients, reduced the possibility for human error, and was more efficient than previous ways of working. However, initiation of the scheme was more achievable in the single site of the hospital in comparison to disparate community pharmacy organisations. Community and hospital pharmacists and organisational leaders will need to work individually and collectively if Refer-to-Pharmacy is to become more widely embedded across health settings.

## Background

The transition from one care setting to another is known to be a time of high risk for preventable medication errors [1–3]. Major changes are often made to patients’ medication regimens during their hospital stay and findings suggest that up to 40% of medications may be discontinued during hospitalisation, while 45% of medications prescribed at discharge are new medications [1]. Hospital discharge is a complex process, involving multiple groups operating in various healthcare settings [4] and poor communication between these settings continues to be a common contributing factor to failures in patient safety [5, 6]. Consequently, the transfer of care from hospital to the community has been identified as a time when miscommunication and unintended changes result in significant risks for patients [7]. It is essential that patients discharged from hospitals have the information and support they need to take their medicines as intended. However, a lack of formal communication channels between hospitals and community settings [8] exemplifies the problems of knowledge sharing across organisational and occupational boundaries within complex health care systems [4]. This lack of knowledge sharing has the potential to contribute to post-discharge medication errors and confusion regarding appropriate discharge medication [9].

Prevention of medication errors by improving transitions of care has become a high priority worldwide [10–12]. Evidence suggests that many errors result from poor ‘handoffs’ between health professionals and inadequate access to clinical records [13]. There is increasing evidence to suggest that information technology (IT) systems, including improved electronic access to patient information, have the potential to reduce medication errors and improve patient safety [14]. However, while some IT innovations in healthcare have failed, or been poorly integrated into existing clinical practices [15–17] the potential for IT innovation to improve healthcare services can only be achieved and maintained through the efforts of those involved in implementing new systems [18]. There is a lack of robust research on the implementation of new technologies, consequently it is vital that future health technologies are evaluated throughout all stages of the technology’s life cycle to understand which factors are likely to maximise successful implementation [19].

Studies exploring the effectiveness of pharmacy interventions to improve transitions of care involving medicines have produced mixed results. While some studies have concluded that involving community pharmacists in transitions of care could reduce unintentional discrepancies with medication, reduce hospital readmissions and provide measureable patient benefit [20–25], other studies investigating the effectiveness of discharge plans have found no significant differences in any of the primary (hospital readmission) or secondary outcomes [26, 27]. In the majority of the studies to date, the discharge information transmitted to the community pharmacist was paper-based; in some cases the patient took the discharge letter to the pharmacist, in other cases it was faxed to the pharmacist. In the UK, the Royal Pharmaceutical Society published guidelines on the transfer of care and highlighted the potential of technology to improve transfer of information between hospital and community pharmacies [10]. However, the report also recognised significant barriers to implementing technological solutions, such as, incompatibilities between IT systems, resistance to change, and engaging staff on the ground. A number of ‘early adopter’ schemes in the UK have recently used varying technological methods of sharing discharge information between hospital and community pharmacy to improve transfer of care [28]. Such schemes are clearly in their infancy and warrant further evaluation to understand how they become embedded and sustained in practice and what promotes or inhibits their implementation.

### The refer-to-pharmacy scheme

One such scheme, and the focus of this paper, is ‘Refer-to-Pharmacy’. Refer-to-Pharmacy is an electronic referral tool, developed in a National Health Services (NHS) Trust based in a large urban town in the North of England and launched in December 2015, which allows hospital pharmacists and pharmacy technicians to electronically refer patients directly to a community pharmacist for the purposes of a post-discharge consultation. Refer-to-Pharmacy is an e-referral service, under which patients who are being discharged from hospitals in one health Trust (1000 beds over five hospitals) are directly referred to a community pharmacy for a post-discharge New Medicines Service (NMS), Medicines Use Review (MUR), or referral into other appropriate care pathways. An MUR is an opportunity for a patient to talk with a community pharmacist about their existing medicines, or changed medicines for long-term conditions, to identify and deal with any associated issues or problems [29]. An NMS is offered by community pharmacists to people starting a new medicine for asthma/chronic obstructive pulmonary disease, type 2 diabetes, hypertension or antiplatelet/anticoagulant treatment to help improve their adherence with medicines [30]. The Refer-to-Pharmacy scheme is not targeted at particular high risk groups, but is designed for any patient who is discharged on one or more medicines (except analgesics and antibiotics). The scheme formalises the referral process, by asking patients to consent prior to discharge and places the onus of following up the referral on the community pharmacist rather than the patient. Under the scheme, a community pharmacist would be expected to have a consultation with the patient, usually within two weeks of being discharged from hospital, with the aim of identifying and remedying any problems with medication. An important element of the scheme is that community pharmacists receive the electronic referral directly, along with the patient’s hospital discharge information which includes details about medication changes. This information has previously been only distributed to the patient’s general practitioner (GP) and not usually provided to community pharmacists.

The analytical approach used to understand the process of implementation was Normalisation Process Theory (NPT) as this was specifically designed to evaluate how complex interventions are implemented, embedded, and sustained [31]. NPT focuses on what people do to contribute to the integration of innovations in their social context and is based on the assumption that interventions become routinely embedded in their organizational and professional contexts as the result of people working, individually and collectively, to implement them. NPT proposes that if participants do not understand, support, or consider an intervention worthwhile, or compatible with their existing work, then the likelihood of successfully integrating new technologies and interventions into the working lives of end users and potential recipients is reduced [18]. Through the lens of NPT, this study seeks to understand the implementation and everyday use of this novel technology and its implications for working practices by understanding the perceptions of the different groups involved in implementing the new intervention and considering the contexts where it was being implemented.

## Methods

### Study design and setting

A qualitative research design using semi-structured interviews was used to explore the experiences and perceptions of hospital pharmacists (*n* = 11), hospital technicians (*n* = 10), and community pharmacists (*n* = 5) involved with Refer-to-Pharmacy, who were involved with early implementation of the scheme during 2015. NPT [32] was used in the design of the interview schedule which was based on the NoMAD instrument (Normalization Measure Development) and adapted this for interviews with community and hospital pharmacy staff (see Table 1) [33]. The NoMAD instrument was designed to measure implementation processes and can be adapted to understand participants’ views on new interventions and their experiences of how an intervention affects their work. The interviews included open ended questions to gather opinions of the scheme and specifically explored factors that promoted or inhibited the implementation of Refer-to-Pharmacy in order to provide detailed insights on how effectively the intervention was embedded and integrated in practice.

**Table 1:** Domains of NPT and related interview questions

Domains	Sub-domain questions
Coherence: refers to how individuals and groups “make sense” of an intervention when they are tasked with implementing a new way of working.	How does Refer-to-Pharmacy differ from what you did before?
	Do participants have a shared understanding of the purpose of Refer-to-Pharmacy?
	How does using Refer-to-Pharmacy affect your work?
	Do you think Refer-to-Pharmacy has the potential to improve medication use for patients?
Cognitive participation: is the relational work people undertake to legitimise and sustain an intervention.	Who drives the scheme forward and gets others involved?
	Do you think that using Refer-to-Pharmacy is a legitimate part of your role?
	Do you have to work with people in different ways in order to deliver the service? Has Refer-to-Pharmacy improved communication between community and hospital pharmacists?
	Will you continue to support the scheme?
Collective action: is the operational work that people do to enact a new intervention.	How does delivering the scheme fit in with everything else you have to do? How long does a typical referral take?
	Do you think pharmacists and technicians are equally able to refer patients?
	Is sufficient training provided to enable you to use the system and identify who is eligible for a referral?
	Are sufficient resources available to support Refer-to-Pharmacy?
Reflexive monitoring: is the appraisal work the people do to understand and evaluate whether the new ways of working are worth sustaining.	Do you get feedback about the referrals you make?
	Do patients think Refer-to-Pharmacy is worthwhile?
	Do you adapt how you use Refer-to-Pharmacy?

Adapted from the NoMAD instrument (Finch et al. 2015)

The Refer-to-Pharmacy scheme was launched in December 2015 and evaluation of early implementation was carried out between February and March 2016. All thirty seven pharmacists and forty four pharmacy technicians in the hospital were participating in the Refer-to-Pharmacy service were approached by email and invited to take part in an interview which was arranged at their convenience. Twenty one interviews with hospital pharmacy staff took place in person or over the telephone if this was more convenient. Similarly, twenty community pharmacists who were participating in the scheme, and had received referrals since the launch of the scheme, were invited to take part. Five community pharmacists agreed to take part in telephone interviews. All interviews were digitally recorded, transcribed verbatim, and uploaded into the NVivo 10 data management software [34].

### Data analysis

The majority of participants were women (22 women and 4 men) and ranged in age from 24 to 64. Interviews were analysed qualitatively in two stages involving an initial thematic analysis and a subsequent interpretation using the four domains of NPT as a framework. This framework was used to explore the factors which were identified as facilitating or impeding the process of implementing the electronic referral scheme. This approach was chosen as it allowed the framework to be practically applied to the data, while allowing for themes to emerge from the data using inductive coding. The research team was comprised of a psychologist, a social scientist and a senior academic pharmacist. Independent analysis of the transcripts was undertaken by two members of the research team and then discussed at collaborative meetings with all authors to evaluate interpretations and reduce bias.

## Results

What follows is an interpretation of the findings using the four constructs of NPT:

### Coherence

In terms of making sense of the new scheme, the combination of the shared understanding of the Refer-to-Pharmacy scheme, with the potential benefits for the patients, and the distinct improvement on previous ways of working meant that coherence was strong in the hospital setting.

*So it’s really quick and easy to use and I don’t have to spend half an hour in a queue, telephone queue…‘Cause I’m comparing it to what I’ve done in the past. I find it much easier. (Int 10, Hospital Pharmacist).*

Refer-to-Pharmacy was considered an improvement on previous practices in both community and hospital settings as it was more efficient than the lengthy process of communicating via telephone and less open to error in that changes were not being transcribed over the telephone. For community pharmacies, the discharge letter was a resource that was previously not always available to them or time consuming to obtain.

*I think in a lot of respects it’s going to save time being wasted and spent chasing things up when we are trying to get the right medication out to the people and to our patients…I think it’s going to save time, it’s not going to waste time. (Community Pharmacist 3).*

Changes to working practices were considered acceptable as they were more time efficient than previous practices and all participants felt that the scheme would improve medication use for patients.

*First it is very timely, you know, when our patients get discharged you get the letter very quickly, the discharge note. Pharmacies are used to patients bringing it to us or the doctor, let us know, that takes time maybe a week or two weeks, in the meantime they may be on the wrong medication or old medication. So, the second is NMS is straightaway, if they are put on some new medication… so the patient understands what they are taking and why they are taking it. (Community Pharmacist 4).*

### Cognitive participation

In terms of the relational work participants undertook to legitimise and support Refer-to-Pharmacy, cognitive participation was not as evident in the community when compared to the hospital setting; where strong leadership meant that staff were motivated to invest time and energy in the scheme.

Implementation was more challenging outside of the controlled and contained environment of a single hospital trust; not all of the community pharmacies that hospital pharmacy teams were referring patients to were engaged in the scheme, and while this was being addressed by the Local Pharmaceutical Committee (LPC), the implications were a potential loss of confidence in the scheme by participants based at the hospital. Practical and logistical difficulties, such as, the ability to gather together all those who would be delivering the scheme for information and training sessions meant that participants in the community were not as well informed.

*As a company I don’t think we’ve talked about Refer-to-Pharmacy at all. I think it’s really, really bad…I think it needs to be backed by our head office…All the LPC [Local Pharmaceutical Committee] meetings when they do those meetings they’ll kind of push them to remind us of certain things like that, but again how many people access those meetings and go to those meetings – it needs like a bigger voice. (Community Pharmacist 3).*

*It’s potentially good but I think it needs to be fully functional in the community first. Maybe not first but they need a really good grounding and understanding and be well informed earlier, before we roll out in the Trust. (Int 4, Hospital Pharmacist).*

Community pharmacy lacked the same initiating leadership in terms of driving the scheme forward and some participants in community pharmacy settings perceived that not all organisational leaders in community pharmacy settings would regard Refer-to-Pharmacy as legitimate part of their remit, as MUR and NMS targets were already being achieved.

*I think the main hold up will be the same. As I said, time management and because, as I say, they are hitting the target of NMS and MUR without this thing, you know, like just from walk in customers. So, you know going into this a little bit more putting effort in, so, you know, they might not be willing to do this. (Community Pharmacist 4).*

Participants in the community described how having access to the discharge letter had improved their communication with GPs as having the discharge information meant they were able to identify and highlight medication discrepancies, which participants perceived was valued by GPs.

*Before Refer-to-Pharmacy there was no point [speaking to the GP], you were just saying you don’t know what has been changed they only depend on the doctor whether they are responsible, you just check, you know, tick the box and that is it. But, now you are a little bit more responsible, you know because you have got the discharge note and you know it shouldn’t be there. So, you just take it on with that, but before it wasn’t, you know, it was just patient and doctor, whether anything changed was their problem. (Community Pharmacist 4).*

### Collective action

In terms of the operational work participants undertook, Refer-to-Pharmacy was easily integrated into existing work in both hospital and community settings as it was regarded as more efficient than previous ways of working. The technology was perceived as self-explanatory and easy to use by all participants, and both pharmacists and technicians in the hospital setting were equally able to refer patients at different points during their hospital stay, meaning that patients were more likely to benefit from the service.

*It’s kind of equal responsibility [between pharmacist and technicians]…you’re not duplicating work because you can see clearly, oh yes, they’ve already done it anyway. So it’s just when they’re captured, whether they’re at admission or at discharge. (Int 12, Hospital Technician).*

### Reflexive monitoring

In terms of evaluating the scheme and determining whether it was worth sustaining, reflexive monitoring was the weakest component of normalisation. While all participants perceived that the scheme was worthwhile and potentially beneficial to patients, a lack of feedback about the impact and outcome of referrals for patients, particularly in the hospital setting, meant that staff were unsure how engaged community pharmacists were with the scheme and how Refer-to-Pharmacy impacted patients. The outcome of referrals was regarded as the missing piece of the jigsaw, and feedback from community pharmacy on how the scheme benefitted patients was perceived as important in sustaining the scheme.

*You would like to know that actually something does happen with them; it [the referral] doesn’t just go into a black hole. (Int 2, Hospital Pharmacist).*

## Discussion

This study is the first to examine perspectives from community and hospital pharmacists about the Refer-to-Pharmacy scheme at an important time in the development of transfer of care initiatives [10]. Previous research has highlighted that implementation of new technologies is dependent on the successful integration into existing practices combined with the collective effort of those involved [18, 19]. This study has extended this understanding by detailing the early stages of the implementation of Refer-to-Pharmacy in a hospital trust and community pharmacies. Participants had a shared understanding of the aims, objectives and potential benefits of the scheme. Participants were prepared to invest time and energy in the scheme, and believed it promoted work, saved time, and reduced the possibility of human error. However, the findings suggest that strong leadership both in the hospital and community settings was necessary to initiate and embed the intervention. While the manageable and contained hospital environment meant initiation of the scheme was achievable, the more disparate environment of community pharmacy may require additional consideration and effort in order to establish greater buy in and support from the varied and separate organisations. Communication about the scheme needed to be a two-way process, and not just from hospital to community pharmacy, as hospital participants were unclear about the impact of the scheme and suggested that feedback was important to maintain interest and motivation. However, this has recently been addressed with the introduction of an e-mail auto-generated by the system when a referral is completed by a community pharmacist. The e-mail summarises the outcome and shows whether there was a prescribing error identified on the prescription, and how much time and medicines waste was saved, if any.

Recently, a systematic review of interventions to improve medication safety called for rigorous evaluation at all stages of an intervention prior to large-scale implementation [25]. Transfer of care schemes involve a number of steps in the process and are influenced by a range of factors at both the individual and organisational level. Understanding opinions, perspectives and how knowledge is shared across organisational and occupational boundaries within complex health care systems is essential for determining factors that promote or inhibit the early stages of implementation. Understanding the socio-technical factors involved in this novel intervention, that not only involves pharmacy input but the use of technology, will provide valuable information to others considering adopting or developing similar transfer or care schemes. For those considering adopting this or similar schemes in their health economy, findings from this study suggest that considerable groundwork should first take place in the community setting to ensure that community pharmacies are engaged in the scheme to ensure that patient referrals are acted upon.

### Future research

Findings from this research will be useful for other healthcare providers who are considering adopting similar technologies. Interventions such as these should undergo rigorous evaluation at all stages prior to large-scale implementation [25]. Most importantly, future research should consider whether patients in receipt of the service are less likely to be readmitted to hospital due to medication related problems. A longitudinal examination of the impact of the service would help to track the impact of the service on readmissions and changes to work practices as the intervention was embedded into existing practice. This would provide further useful information for others who are considering implementing the service. Future studies should also consider the patient perspective, including the impact of the service on the patient and whether patients feel that Refer-to-Pharmacy has helped them to better understand their medicines.

### Limitations

Due to the nature of qualitative research, findings may be of limited generalisability and findings in other contexts and settings may differ. The hospital where this study was carried out was the first Trust to introduce this service, and while valuable lessons can be learnt from this single case study, it is important to bear in mind that how this scheme was implemented in this context may differ in other hospitals and community pharmacies. Furthermore, while there was inclusion of most pharmacists and technicians delivering the service in the hospital setting, it may be likely that those community pharmacists who agreed to take part in the interviews were more engaged and positive towards the scheme than those who did not volunteer to be interviewed. Interviews were carried out at the early stages of the scheme being introduced; while it is recommended that schemes such as these be evaluated at all stages, how people work to implement the scheme is likely to change over time.

## Conclusion

Shared views on the perceived benefits of Refer-to-Pharmacy and ease of integration into existing work practices were key factors that promoted the implementation of the scheme in both the community and hospital pharmacy settings. Barriers to implementation were more evident in the community setting where it was more challenging to promote and legitimise the scheme due to the disparate nature of community pharmacy. Community and hospital pharmacists and organisational leaders will need to work individually and collectively if Refer-to-Pharmacy is to become routinely embedded across other healthcare settings.

## Abbreviations

CPPE: Centre for Pharmacy Postgraduate Education
GP: General practitioner
LPC: Local Pharmaceutical Committee
MUR: Medicines Usage Review
NHS: National Health Services
NMS: New Medicines Service
NoMAD: Normalization Measure Development is instrument designed for assessing the implementation of complex interventions
NPT: Normalisation Process Theory
